# Decadal trends of the upper ocean salinity in the tropical Indo-Pacific since mid-1990s

**DOI:** 10.1038/srep16050

**Published:** 2015-11-02

**Authors:** Yan Du, Yuhong Zhang, Ming Feng, Tianyu Wang, Ningning Zhang, Susan Wijffels

**Affiliations:** 1 State Key Laboratory of Tropical Oceanography, South China Sea Institute of Oceanology, Chinese Academy of Sciences, Guangzhou, China; 2 CSIRO Oceans and Atmosphere Flagship, Floreat, Western Australia, Australia; 3 College of Physical and Environmental Oceanography, Ocean University of China, Qingdao, China; 4 CSIRO Oceans and Atmosphere Flagship, Hobart, Tasmania, Australia

## Abstract

A contrasting trend pattern of sea surface salinity (SSS) between the western tropical Pacific (WTP) and the southeastern tropical Indian Ocean (SETIO) is observed during 2004–2013, with significant salinity increase in the WTP and freshening in the SETIO. In this study, we show that increased precipitation around the Maritime Continent (MC), decreased precipitation in the western-central tropical Pacific, and ocean advection processes contribute to the salinity trends in the region. From a longer historical record, these salinity trends started in the mid-1990s, a few years before the Global Warming Hiatus from 1998 to present. The salinity trends are associated a strengthening trend of the Walker Circulation over the tropical Indo-Pacific, which have reversed the long-term salinity changes in the tropical Indo-Pacific as a consequence of global warming. Understanding decadal variations of SSS in the tropical Indo-Pacific will better inform on how the tropical hydrological cycle will be affected by the natural variability and a warming climate.

As an indicator of the global hydrological cycle, ocean salinity is an essential measure of the large scale climate variability^1^. The spatial distributions of the sea surface salinity (SSS) in the global ocean well reflect the pattern of air-sea freshwater fluxes, the evaporation and precipitation^2^. Previous studies revealed that the western tropical Pacific (WTP) had been freshening since 1950s, as the most significant salinity change over the global ocean^3^,^4^. It had resulted from an increase of global hydrological cycle in a global warming scenario, a so called “wet get wetter” or “dry get drier” response^5^,^6^,^7^,^8^, constrained by the Clausius-Clapeyron relationship^6^,^9^.

However, a remarkable hiatus of the global warming has occurred since the beginning of the 21^th^ century^10^, which is tied to sea surface temperature (SST) cooling in the eastern equatorial Pacific and intensification of the Walker Circulation^11^,^12^,^13^. The corresponding strengthening trend of the Pacific trade winds has caused rapid sea level rise in the western tropical Pacific and the thermocline shoaling in the east, as well as a strengthening trend of the Pacific subtropical cells^14^,^15^. Despite the research progresses in the regional climatic impacts of the global warming hiatus, its influences on the global hydrological cycle are still not well studied.

Historically, reliable ocean temperature and salinity observations are derived from sparse ship-board samplings or limited mooring observations^16^. Over the past decade, the Array for Real-time Geostrophic Oceanography (Argo) program has significantly increased the *in situ* temperature and salinity observations^17^. With a near global coverage, Argo program has allowed an assessment of salinity variability in upper ~2000 m in the global oceans.

Using the Argo salinity observations as well as historical data resources, this study investigates the salinity trends in the tropical Indo-Pacific over the past decade (2004–2013). We have identified an upper ocean salinity increase trend in the WTP and a freshening trend in the southeastern tropical Indian Ocean (SETIO), and attributed them to an intensified Walker Circulation from mid-1990s.

## Results

### Surface salinity trends and intensification of Walker Circulation

**Figure 1:**
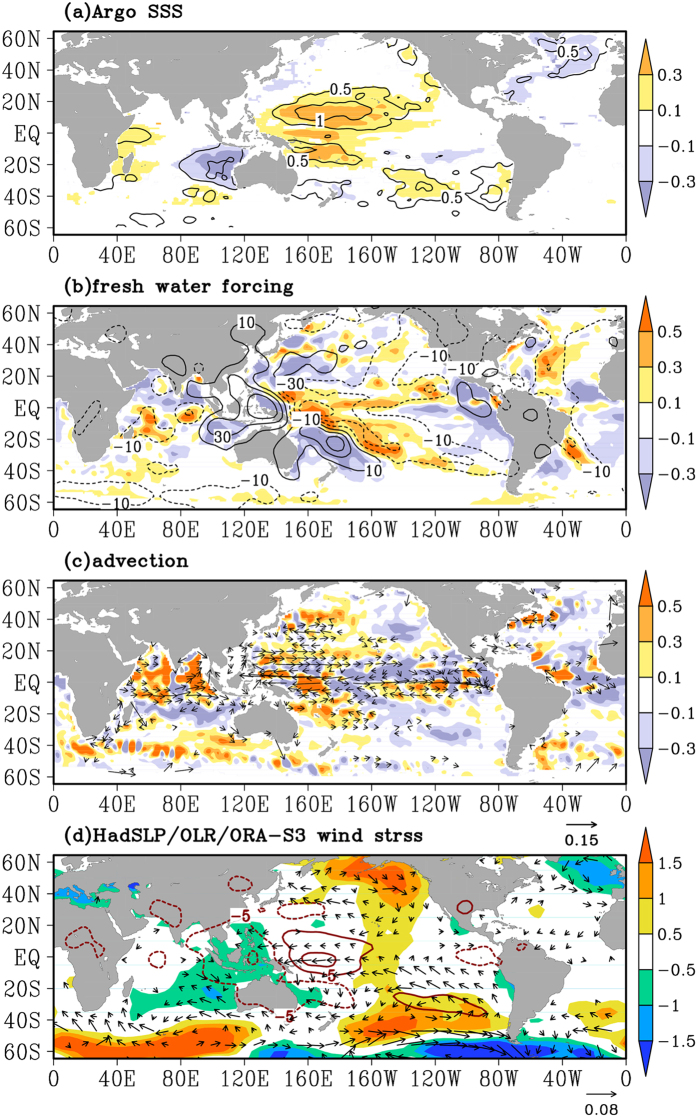
Linear trends of SSS, atmospheric fields, and oceanic advection. **(a**) SSS in the Argo data (shaded, psu·10 year^−1^), superimposed with ratio between standard deviation of the trend and the detrended SSS (contours). The linear trend is not significant at the 95% confidence level are not shown. (**b**) Fresh water forcing (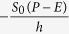, shaded, psu·year^−1^·10 year^−1^), and GPCP precipitation (contours, mm·month^−1^·10 year^−1^). (**c**) Salinity advection (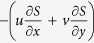, shaded, psu·year^−1^·10 year^−1^), and the OSCAR ocean surface currents (vectors, m·s^−1^·10 year^−1^). (**d**) Sea level pressure (shaded, pa·10 year^−1^), outgoing longwave radiation (contours, w·m^−2^·10 year^−1^), and ORA-S3 wind stress (vectors, kg·m^−1^·s^−2^·10 year^−1^). All the trends are presented with linear changes for the period of 2004–2013. The figure is generated using GrADS.

The WTP becoming saltier and the SETIO becoming fresher are the dominant features in the linear trends of SSS from Argo float data in the global ocean during 2004–2013 (Fig. 1a). Comparing standard deviations of the observed trends with the detrended SSS variability, the Indo-Pacific warm pool stands out as the region with the most robust SSS trends in the past decade. An Empirical Orthogonal Function (EOF) analysis also verifies that the contrasting trend pattern between the WTP and SETIO is the dominant salinity signal in the Indo-Pacific over the past decade (Supplementary-Fig. 1). This pattern is clearly reflected in the linear trends of the surface freshwater forcing, evaporation minus precipitation, in which precipitation trend is a dominant contributor on decadal time scales (Fig. 1b). During the same period, the horizontal advection of SSS, characterized with greater spatial variations, contributes to part of the positive SSS trends in the WTP, and tends to enhance and spread the salinity change to a broad area in the SETIO (Fig. 1c).

Atmospheric fields show that the SSS changes correspond to an intensification of trade winds in the tropical Pacific, which is a good indicator of the strength of the Walker Circulation over the Indo-Pacific equatorial ocean (Fig. 1d). Sea level pressures drop over the Maritime Continent (MC)-SETIO and increase over the western-central tropical Pacific, accompanying with the strengthening trade winds in the western equatorial Pacific (Fig. 1d). Contrasting trends of outgoing longwave radiation (OLR) between MC-SETIO and WTP, a proxy for convective precipitations, are consistent with the precipitation change over the region (Fig. 1d).

### Ocean circulation and thermocline adjustment

**Figure 2:**
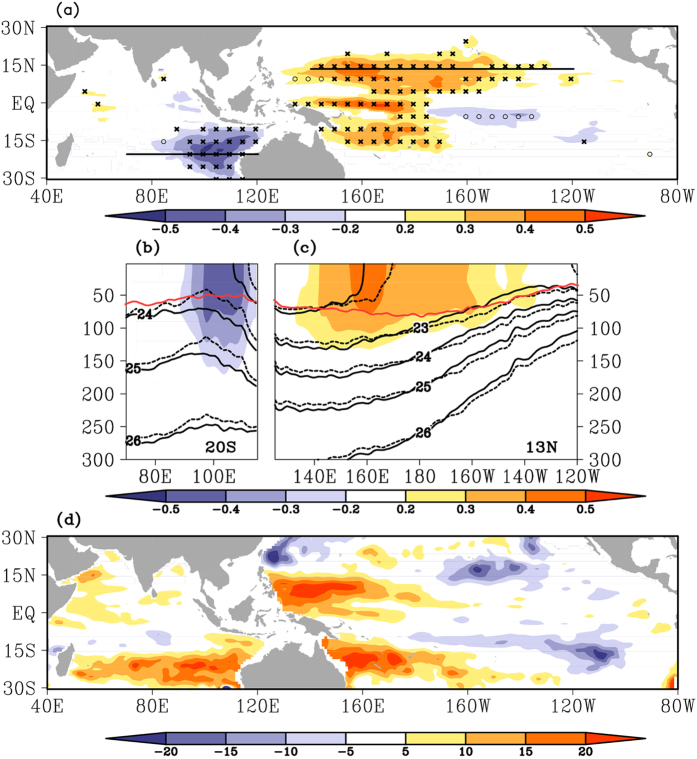
Linear trends of salinity in vertical and thermocline depth. (**a**) The maximum salinity trends (shaded, psu·10 year^−1^) in the upper 300 m and their corresponding depths (x: <50 m; o- >100 m). (**b**) Salinity trends (shaded, psu·10 year^−1^), potential density (black contours: solid lines present for 2011–2013 average and dashed lines for 2004–2006 average, kg/m^−3^), and climatological annual mean mixed layer depth (red curve, m) along the 20° S section in the southeastern tropical Indian Ocean. (**c**) Same as (**b**), but along the 13° N section in the western tropical Pacific. (**d**) Linear trends of the depth of 22 °C isotherm (shaded, m). All trends are for the period of 2004–2013. The linear trend of salinity is not significant at the 95% confidence level are not shown. Two horizontal lines in (**a**) indicate the locations of the sections in (**b**) and (**c**). The figure is generated using GrADS.

The maximum linear salinity trends, with amplitude larger than 0.4 psu, are found mostly in the upper 50 m layer (Fig. 2a), the average mixed layer depth in the Indo-Pacific warm pool, suggesting dominant influences from the fresh water flux forcing at the air-sea surface (Fig. 1b). A maximum freshening area in the central equatorial Pacific occurs over 100 m depth, likely reflecting the thermocline shoaling, where the low salinity water underneath originates from the subtropical Pacific mode water^18^.

In the northwestern Pacific at 13° N, the layer of high salinity trends greater than 0.2 psu could reach 150 m depth around 160° E, and become shallower in the east following the tilt of the thermocline (Fig. 2c). The zonal variation of thermocline depth, comparing the averages in the first and last 3 years during the 2004–2013 period, changes sign at 180° E, deepening in the west and shoaling in the east. The trends of thermocline depth (represented by the 22 °C isotherm, Z22) show a 15–20 m deepening in the WTP and a slight shoaling in the eastern tropical Pacific (Fig. 2d), consistent with sea level trends over the region during the same period^15^, reflecting the impact of strengthening trade winds (Fig. 1d). The changes of thermocline depth accompany with the changes in the ocean circulation. The Pacific tropical circulation contracts in latitudinal extent, the bifurcation latitude of the North Equatorial Current (NEC) moves southward, and thus the subtropical circulation expands in the recent decade^19^,^20^ (Fig. 1c), which causes the positive surface salinity trends in particular region of the WTP, especially in the salinity front zone regions.

Along a section west of Australia at 20° S in the Indian Ocean, the layer of significant freshening trends reach 150 m depth, and the thermocline deepens by about 20 m (Fig. 2b,d). Recent studies indicate that the decadal variations of SSH/thermocline in the southeastern Indian Ocean origins from the WTP through the wave guide in the Indonesian Seas^21^,^22^. Besides, in the exit region of the Indonesian Throughflow (ITF), the convergence induced by the ocean circulation could deepen the thermocline in the SETIO^23^. With a weakening South Equatorial Current (SEC) in the Indian Ocean (Fig. 1c), and a strengthening ITF recorded by an ADCP current mooring at the Makassar Strait^24^, fresh water converges in the SETIO and deepens the thermocline (Fig. 2b,d).

### Decadal variations from a longer salinity record

**Figure 3:**
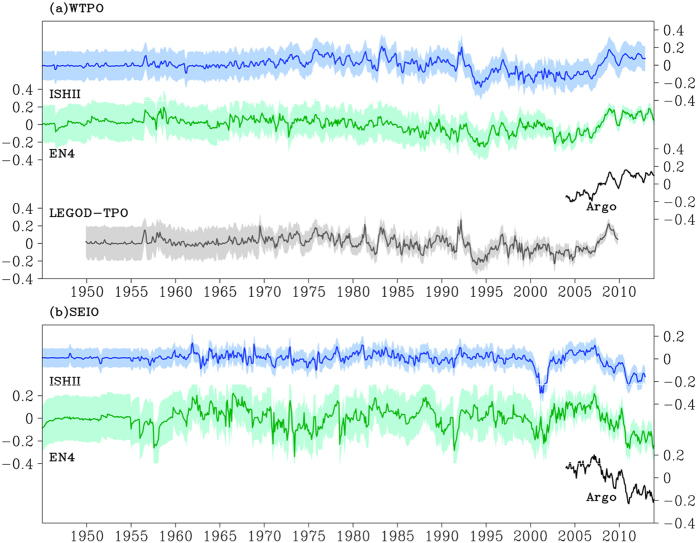
Regional averaged SSS anomalies time series from 1945 to 2013 (psu). (**a**) The western tropical Pacific (140°–200° E, 20° S-20° N). (**b**) The south eastern tropical Indian Ocean (90°–120° E, 25°–5° S) derived from different data products. Shadings on the lines indicate the error bar 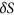, which are provided with the salinity data in LEGOD-TPO and EN4. The 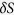 in ISHII is calculated by 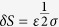. Where 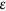 is the normalized error which is provided with the salinity data, and 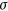 is the standard deviation of SSS anomaly. The figure is generated using GrADS.

Previous studies identified that the upper ocean salinity decreases in the WTP and increases in the SETIO from 1950s to 1990s/early 2000s^3^,^4^,^25^,^26^,^27^. Obviously, the observed SSS trends in the recent decade are opposite to the early period. To examine the long term change, the SSS anomalies averaged in the SETIO and WTP are shown in Fig. 3. Due to the scarcity of the T/S profile observations prior to the 1970s (Supplementary-Fig. 2)^26^, and the salinity time series lack the natural variability as observed in recent decades, so we do not discuss the result before the 1970s.

All the salinity datasets have consistent variations since the mid-1990s. They show a robust upward trend of SSS in the WTP and downward trend in the SETIO. Before that, there are discrepancies among different products (Fig. 3). Considering the difference in original data sampling and processing methods, only the common decadal features are discussed here. In the WTP, SSS freshens from 1970s to mid-1990s, despite strong interannual variability. The freshening trend is likely attributed to both the strengthened hydrological cycle associated with the global warming^4^,^26^ and the PDO-induced Walker circulation weakening from mid-1970 to mid-1990^28^,^29^. The former induces the SSS decrease within the tropical rainfall band, which is consistency with the “wet-get-wetter” mechanism^5^,^6^,^7^,^8^. The latter enhances the SSS decrease in the WTP, making it more robust than the SSS change in the SETIO. A slow oscillation of sea surface salinity occurs from mid-1990s to mid-2000s, which is not well understood so far. A recent study suggested that it is probably due to the zonal advection and precipitation related with the El Niño-Modoki and La Niña events^30^. In the SETIO, the interannual variability dominates the SSS variation before 2004. A recent event is the significant freshening in 2000–2002, due to extreme fresh water flux during a long lasting La Niña event^31^. Thus, from historical perspective, the recent SSS changes do not represent a monotonic trend but a part of the decadal variability.

### Decadal shift in 1990s and Global Warming Hiatus

**Figure 4:**
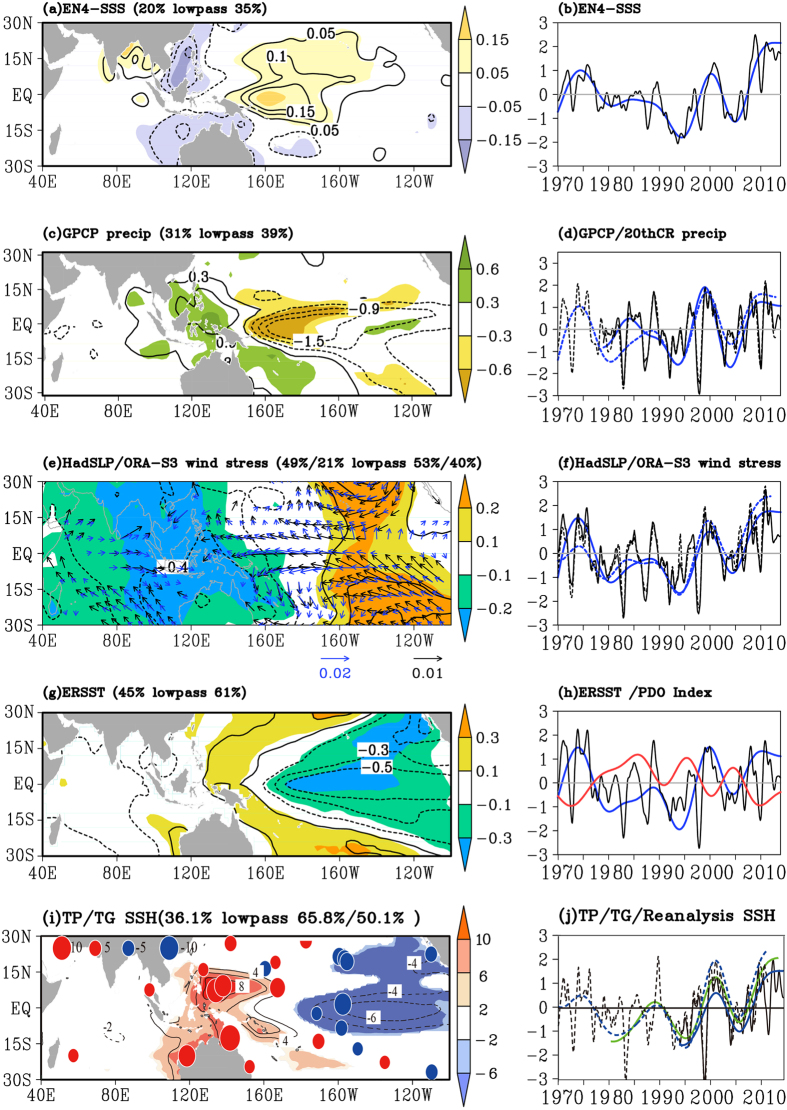
Spatial loadings of the first EOF modes (left column) for monthly SSS anomalies (contours) and their low-frequency variability (shaded), and their principal components (right column) for the monthly anomalies (black curve) and the low-frequency variability (blue curve). (**a**) SSS (psu). (**c**) Precipitation (mm·month^−1^). (**e**) Sea level pressure (pa), superimposed with wind stress (black vectors for anomaly and blue vectors for the low-frequency variability, kg·m^−1^·s^−2^). (**g**) SST ( °C). (**i**) AVISO SSH (cm), superimposed with sea level from tide gauges (solid circle for the low-frequency variability). (**b**,**d**,**f**,**h**, and **j**) are principal components of (**a**,**c**,**e**,**g**, and **i**), respectively. The 20^th^ century reanalysis precipitation anomaly (dashed black curve) and its corresponding low-frequency variability (dashed blue curve) are superimposed in (**d**). Wind stress anomaly (dashed black curve) and its corresponding low-frequency variability (dashed blue curve) are superimposed in (**f**). The low-frequency variability of PDO index (red curve) is superimposed in (**h**). Reconstructed sea level anomaly (dashed black curve) and its low-frequency variability (dashed blue curve), and the low-frequency variability of sea level from tide gauges (solid green curve) are superimposed in (**j**). The figure is generated using GrADS.

An EOF analysis is applied to the UK Metoffice-EN4 SSS and associated variables in atmosphere and ocean in the tropical Indo-Pacific (Fig. 4). The first EOF mode (EOF1) of the SSS shows contrasting variations between the South China Sea (SCS)-SETIO and the WTP, high in the east and low in the west, explaining 20%  of the total root mean square (RMS) variance, which increase to 35%  with only low-frequency signals considered (Fig. 4a). This pattern mainly reflects a La Niña-like feature^32^. The absence of SSS signal in the SCS in Fig. 1a is due to the lack of Argo floats in the region (Supplementary-Fig. 3). A recent study reported that the SCS reaches the freshest status in 2012 during the recent decade, due to enhanced fresh water flux^33^. Note the weak signal in the Indonesian Seas in Fig. 4a might also be due to the lack of the *in situ* observations (Supplementary-Fig. 3).

The first principle component (PC1) generally agrees well with the SSS time series averaged in the WTP (Figs 3a and 4b), but manifests a significant shift in long term trend in the mid-1990s: freshening before 1993 and becoming saline after that, superimposed on the interannual oscillations (Fig. 4b). Note the spatial pattern of the SSS trends in the two epochs are not symmetrical (Supplementary-Fig. 4), which partly explains the low explained variance.

Precipitations also indicate the shift in mid-1990s (Fig. 4c,d, and Supplementary-Fig. 4), which explains the shift in SSS, though the ratio between standard deviation of the trend and the detrended precipitation is much smaller. The global climate system bear a dramatic change in late-1990s/early-2000s, with the mean global surface air temperature ceases its long term warming trend in recent 16 years, being called the Global Warming Hiatus^10^. Being a few years earlier, the shift of SSS trends in mid-1990s reflects the same change^34^. Note the Walk Circulation has been slowing down before and then intensifying after mid-1990s (Fig. 4e,f and Supplementary-Fig. 4), which has induced a SST cooling in the eastern and central tropical Pacific since mid-1990s (Fig. 4g,h, and Supplementary-Fig. 4). The latter is regarded as the key driver to the Global Warming Hiatus^12^. Recent studies suggest that the SST cooling in the eastern and central tropical Pacific is sustained by the thermocline variability^13^. The SSH variation, the proxy of the thermocline variation in the tropics, featuring an enhanced east low-west high tilt in the tropical Pacific that starts from mid-1990s (Fig. 4i,j, and Supplementary-Fig. 4).

## Discussions

**Figure 5:**
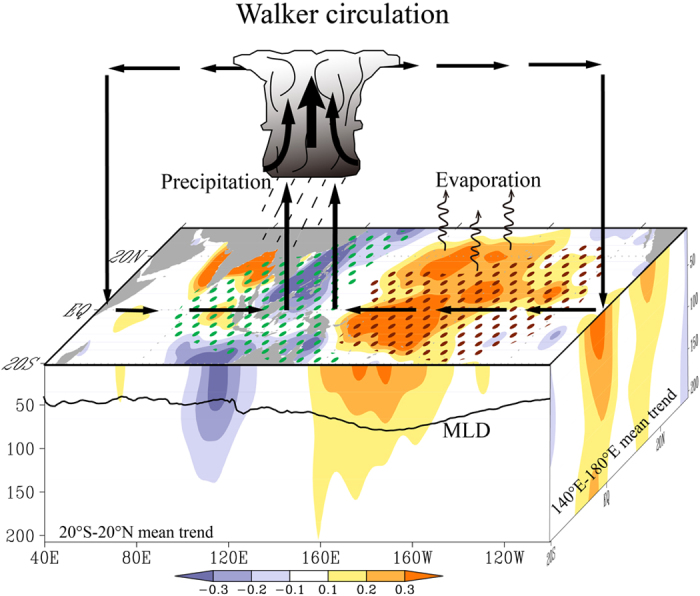
Schematic diagram of the intensification of the Walker Circulation and its impact on the SSS trends since the mid-1990s. The background map (shadings) uses EN4 SSS trends for the period of mid-1990s–2013. The E-P trends are doted in the surface (green: E-P<-20 mm·month^−1^; brown: E-P>20 mm·month^−1^). The mixed layer depth (MLD) is averaged from 20° S to 20° N for the period from 2011to 2013. The figure is generated using GrADS and Adobe Illustrator.

Argo profiles provide unprecedented salinity observations in global coverage. Robust SSS trends in the tropical Indo-Pacific are recorded in Argo since 2004, as part of the decadal trend started from mid-1990s in reconstructed salinity dataset. The atmospheric reanalysis fields reveal that the intensification of the Walker Circulation is a key driver of SSS trends. It enhances the precipitation over the Maritime Continent and adjacent ocean and reduces precipitation over the western-central tropical Pacific, resulting in a salinfication trend in the WTP and freshening trend in the SCS-SETIO (Fig. 5). The intensification of the Walker Circulation is associated with strengthened trade winds in the tropical Pacific, causing changes in ocean thermocline structure and horizontal circulation. The fresh water forcing determines the basin-scale patterns of SSS change, whereas the ocean dynamic processes restructure its spatial distribution. Horizontal advection of ocean currents and ocean convergence favor the salinfication trend in the WTP and affect the strength and spatial distribution of the freshening trend in the SETIO.

The pattern of SSS trend in the recent decade is opposite to that from 1970s to mid-1990s (Supplementary-Fig. 4). Previous studies revealed that the anthropogenic forcing/global warming induce a “wet get wetter” response to the hydrological cycle^5^,^6^,^7^,^8^. The pattern of the change is similar to the climatological mean (Supplementary-Fig. 3). This study found that the natural variability could bring similar even strong change in sea surface salinity and hydrological cycle, with the influence intensified in tropics in decadal time scale. Both natural variability and anthropogenic forcing are important to the sea surface salinity change in the WTP. The recent strengthening trend of the Walker circulation and the SSS trend in the WTP could not be explained by anthropogenic forcing, rather than be part of the natural decadal climate variability of the climate system. It is crucial to understand the relationship between the variability of the Walker circulation and the precipitation anomalies in the Indo-Pacific Ocean, in order to verify to what extent the ocean salinity variability can be used as an indicator of the variability of the global hydrological cycle.

Climate community believes that the SST cooling in the central and eastern Pacific contributes to the Global Warming Hiatus^12^. Considering that the precipitation and the salinity stratification formed barrier layer in the warm pool are important for the Indo-Pacific climate^35^, it is not clear yet if the increase of salinity in the WTP and the freshening in the SETIO would interact with the Walker circulation, and somehow maintains the La Niña-like condition in the Pacific. Sustained ocean observations and sensitivity experiments using coupled ocean-atmosphere models may help to address these questions in the future.

## Methods

### Data

We use the Argo salinity profiles as well as gridded monthly product, the latter provided by the Scripps Institution of Oceanography from 2004 to 2013^17^. The NASA/GSFC Global Precipitation Climatology Project (GPCP) Version 2.2^36^,^37^ is available since 1979. Net fresh water flux is obtained by evaporation minus precipitation (E-P) for the period of 1979–2013, in which the evaporation is derived from the Objectively Analyzed air-sea Flux (OAFlux) dataset^38^. For comparison, the 20^th^ century reanalysis (20CRv2) precipitation is used^39^. Satellite field-derived ocean surface currents, Ocean Surface Current Analysis Real-time (OSCAR), are provided by the NOAA^40^. To calculate the horizontal salinity advection, OSCAR ocean surface currents are used in combination with the Argo salinity gradients. Sea surface height (SSH) uses AVISO merged products (http://www.aviso.oceanobs.com/en). Both of them are available since 1993. Sea level data from tide gauges are obtained from University of Hawaii Sea Level Center (http://uhslc.soest.hawaii.edu) and the sea level reconstructions from NASA JPL/PO.DAAC^41^. The former includes 40 tide gauge stations in the tropical Indo-Pacific (30° E-90° W, 35° S-25° N), most of them recorded from 1980.

We also use wind stress from the ECMWF ORA-S3^42^, salinity from the UK Met Office EN.4.0.2 objective analyses^43^, the ISHII v6.13 analyses^44^, and the French SSS Observation Service^16^ for Tropical Pacific Ocean, Hadley Center Sea Level Pressure (HadSLP2)^45^, and the latest version of NOAA Extended Reconstructed SST (ERSST V3b)^46^. Since the salinity observations with reasonable samplings are only available in recent four decades, we focus on the period of 1970–present. We use monthly products unless otherwise stated.

### Data processing and Empirical Orthogonal Function analysis

Anomalies of the above variables are referenced to each period. All anomalies are filtered in 5-month running window. The low frequency signals are obtained by removing the global means and then filtered with an 84-month lowpass filter.

The Empirical Orthogonal Function (EOF) analysis is used in this study. EOF is a method to extract the main feature vectors from a matrix^47^, which was first introduced into meteorology and climate research by Lorenz^48^, and then was widely used in the ocean and atmosphere research. The EOF analysis is good at decomposing the temporal and spatial signatures, we use it to analyze the SSS, atmospheric fields, and SSH variations during 1970–2013.

### Mixed-layer depth and Mixed-layer salinity budget

The MLD in this study is defined as a difference in potential density from the surface value, which is equivalent to a 0.5 °C decrease in temperature^49^.

The salinity budget within the MLD can be estimated from the following equation^50^,


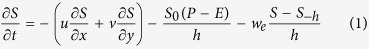


Where 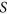, 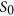, and 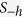 are the mixed-layer salinity, SSS, and the salinity right below the mixed layer. *u* and *v* are the horizontal velocity, *P* and *E* are the precipitation and evaporation rates, *h* is the depth of the mixed layer.

## Additional Information

**How to cite this article**: Du, Y. *et al.* Decadal trends of the upper ocean salinity in the tropical Indo-Pacific since mid-1990s. *Sci. Rep.* **5**, 16050; doi: 10.1038/srep16050 (2015).

## Supplementary Material

Supplementary Information

## References

[b1] Schmitt R. N. W. Salinity and the global water cycle. Oceanography. 21, 12–19 (2008).

[b2] Yu L., Jin X. & Weller R. A. Annual, seasonal, and interannual variability of air-sea heat fluxes in the Indian Ocean. J. Clim. 20, 3190–3209 (2007).

[b3] Boyer T. P. *et al.* Linear trends in salinity for the World Ocean, 1955–1998. Geophys. Res. Lett. 32, L01604 (2005).

[b4] Cravatte S. *et al.* Observed freshening and warming of the western Pacific warm pool. Clim. Dyn. 33, 565–589 (2009).

[b5] Mitchell J. F., Wilson C. A. & Cunnington W. M. On CO2 climate sensitivity and model dependence of results. Q. J. R. Meteorol. Soc. 113, 293–322 (1987).

[b6] Held I. M. & Soden B. J. Robust response of the hydrological cycle to global warming. J. Clim. 19, 5686–5699 (2006).

[b7] Chou C., Neelin J. D., Chen C.-A. & Tu J.-Y. Evaluating the “Rich-Get-Richer” Mechanism in Tropical Precipitation Change under Global Warming. J. Clim. 22, 1982–2005 (2009).

[b8] Bony S. *et al.* Robust direct effect of carbon dioxide on tropical circulation and regional precipitation. Nature Geosci. 6, 447–451 (2013).

[b9] Durack P. J., Wijffels S. E. & Matear R. J. Ocean salinities reveal strong global water cycle intensification during 1950 to 2000. Science 336, 455–458 (2012). 22539717 10.1126/science.1212222

[b10] Meehl G. A. *et al.* Model-based evidence of deep-ocean heat uptake during surface-temperature hiatus periods. Nat. Clim. Change 1, 360–364 (2011).

[b11] Tokinaga H. *et al.* Slowdown of the Walker circulation driven by tropical Indo-Pacific warming. Nature 491, 439–443 (2012). 23151588 10.1038/nature11576

[b12] Kosaka Y. & Xie S.-P. Recent global-warming hiatus tied to equatorial Pacific surface cooling. Nature 501, 403–407 (2013). 23995690 10.1038/nature12534

[b13] England M. H. *et al.* Recent intensification of wind-driven circulation in the Pacific and the ongoing warming hiatus. Nat. Clim. Change 4, 222–227 (2014).

[b14] Feng M., McPhaden M. J. & Lee T. Decadal variability of the Pacific subtropical cells and their influence on the southeast Indian Ocean. Geophys. Res. Lett. 37, L09606 (2010).

[b15] Merrifield M. A. A shift in western tropical Pacific sea level trends during the 1990s. J. Clim. 24, 4162–4138 (2011).

[b16] Delcroix T. *et al.* A gridded sea surface salinity data set for the tropical Pacific with sample applications (1950–2008). Deep-Sea Res. 58, 38–48 (2011).

[b17] Roemmich D. & Gilson J. The 2004–2008 mean and annual cycle of temperature, salinity, and steric height in the global ocean from the Argo program. Prog. Oceanogr. 82, 81–100 (2009).

[b18] Qu T. *et al.* Origin and pathway of equatorial 13 °C water in the Pacific identified by a simulated passive tracer and its adjoint. J. Phys. Oceanogr. 39, 1836–1853 (2009).

[b19] Qiu B. & Chen S. Interannual-to-Decadal Variability in the Bifurcation of the North Equatorial Current off the Philippines. J. Phys. Oceanogr. 40, 2525–2538 (2010).

[b20] Gordon A. L., Flament P., Villanoy C. & Centurioni L. The nascent Kuroshio of Lamon Bay. J. Geophys. Res. Oceans 119, 4251–4263 (2014).

[b21] Han W. *et al.* Patterns of Indian Ocean sea-level change in a warming climate. Nat. Geosci. 3, 546–550 (2010).

[b22] Feng M., Boning C. & Biastoch A. The reversal of the multi-decadal trends of the equatorial Pacific easterly winds, and the Indonesian Throughflow and Leeuwin Current transports. Geophys. Res. Lett. 38, L11604 (2011).

[b23] Qu T. *et al.* Buffering effect and its related ocean dynamics in the Indonesian Throughflow region. J. Phys. Oceanogr. 38, 503–516 (2008).

[b24] Gordon A. L. *et al.* South China Sea Throughflow impact on the Indonesian Throughflow. Geophys. Res. Lett. 39, L11602 (2012).

[b25] Hosoda S., Sugo T., Shikama N. & Mizuno K. Global surface layer salinity change detected by Argo and its implication for hydrological cycle intensification. J. Oceanogr. 65, 579–586 (2009).

[b26] Durack P. J.& Wijffels S. E. Fifty-year trends in global ocean salinities and their relationship to broad-scale warming. J. Clim. 23, 4342–4362 (2010).

[b27] Skliris N. *et al.* Salinity changes in the World Ocean since 1950 in relation to changing surface freshwater fluxes. Clim. Dyn. 42, 709–736 (2014).

[b28] Mantua N. J., Hare, Zhang S. R., Wallace Y., J. M. & Francis, R. C. A Pacific interdecadal oscillation with impacts on salmon production. Bull. Amer. Meteor. Soc. 78, 1069–1079 (1997).

[b29] Deser C., Phillips A. S. & Hurrell J. W. Pacific interdecadal climate variability: Linkages between the tropics and North Pacific during boreal winter since 1900. J. Clim. 17, 3109–3124 (2004).

[b30] Hasegawa T. *et al.* Upper-ocean salinity variability in the tropical Pacific: Case study for quasi-decadal shift during the 2000s using TRITON buoys and Argo floats. J. Cli**m**. 26, 8126–8138 (2013).

[b31] Phillips H. E., Wijffels S. E. & Fing M. Interannual variability in the freshwater content of the Indonesian-Australian Basin. Geophys. Res. Lett. 32, L03603 (2005).

[b32] Qu T. & Yu J.-Y. ENSO indices from sea surface salinity observed by Aquarius and Argo. J. Oceanogr. 70 **(4)**, 367–375 (2014).

[b33] Zeng L. *et al.* Freshening in the South China Sea during 2012 revealed by Aquarius and *in situ* data. J. Geophys. Res. Oceans 119, 8296–8314 (2014).

[b34] Delcroix T., Cravatte S. & McPhaden M. J. Decadal variations and trends in tropical Pacific sea surface salinity since 1970. J. Geophys. Res. 112, C03012 (2007).

[b35] Maes C., Picaut J. & Belamari S. Importance of the salinity barrier layer for the buildup of El Niño. J. Clim. 18, 104–118 (2005).

[b36] Adler R. F. *et al.* The version 2.1 Global Precipitation Climatology Project (GPCP) monthly precipitation analysis (1979–Present). J. Hydrometeor. 4, 1147–1167 (2003).

[b37] Huffman G. J., Adler R. F., Bolvin D. T. & Gu G. Improving the global precipitation record: GPCP Version 2.1. Geophys. Res. Lett. 36, L17808 (2009).

[b38] Yu L. & Weller R. A. Objectively analyzed air–sea flux fields for the global ice-free oceans (1981–2005). Bull. Am. Meteorol. Soc. 88, 527–539 (2007).

[b39] Compo G. P. *et al.* The twentieth century reanalysis project. Q. R. Meteorol. Soc. 137, 1–28 (2011).

[b40] Bonjean F. & Lagerloef G. S. E. Diagnostic Model and Analysis of the Surface Currents in the Tropical Pacific Ocean. J. Phys. Oceanogr. 32, 2938–2954 (2002).

[b41] Hamlington B. D. *et al.* Reconstructing sea level using cyclostationary empirical orthogonal functions. J. Geophys. Res. 116, C12015 (2011).

[b42] Balmaseda M. A., Vidard A. & Anderson D. L. T. The ECMWF Ocean Analysis System: ORA-S3. Mon. Wea. Rev. 136, 3018–3034 (2008).

[b43] Good S. A., Martin M. J. & Rayner N. A. EN4: quality controlled ocean temperature and salinity profiles and monthly objective analyses with uncertainty estimates. J. Geophys. Res. 118, 6704–6716 (2013).

[b44] Ishii M., Kimoto M., Sakamoto K. & Iwasaki S.-I. Steric sea level changes estimated from historical subsurface temperature and salinity analyses. J. Oceanogr. 62, 155–170 (2006).

[b45] Allan R. J. & Ansell T. J. A new globally complete monthly historical gridded mean Sea Level Pressure dataset (HadSLP2): 1850–2004. J. Clim. 19, 5816–5842 (2010).

[b46] Smith T. M. & Reynolds R. W. Extended reconstruction of global sea surface temperatures based on COADS data (1854–1997). J. Clim. 16, 1495–1510 (2003).

[b47] Pearson K. On lines and planes of closest fit to systems of points in space. Phil. Mag. 2, 559–572 (1901).

[b48] Lorenz E. N. Empirical orthogonal functions and statistical weather prediction. Technical report, Statistical Forecast Project Report 1, Dept. of Meteor., MIT, 48pp (1956).

[b49] Kara A. B., Rochford P. A. & Hurlburt H. E. An optimal definition for ocean mixed layer depth. J. Geophys. Res. 105, 16, 803–16,821 (2000).

[b50] Feng M., Hacker P. & Lukas R. Upper ocean heat and salt balances in response to a westerly wind burst in the western equatorial Pacific during TOGA COARE. J. Geophys. Res. 103(C5), 10,289–10,311 (1998).

