# Supplementary material for: Decadal trends of the upper ocean salinity in the tropical Indo-Pacific since mid-1990s
Source: Sci Rep. 2015 Nov 2;5:16050. doi: 10.1038/srep16050 (PMC4629206; doi:10.1038/srep16050)
Supplement: Supplementary Information [file srep16050-s1.pdf]

Supplementary Information for

"Decadal trends of the upper ocean salinity in the tropical Indo-Pacific since mid-1990s"

Yan DU[1], Yuhong ZHANG[1], Ming FENG[2], Tianyu WANG[1], Ningning ZHANG[2,3], Susan WIJFFELS[4]

[1]*State Key Laboratory of Tropical Oceanography, South China Sea Institute of Oceanology, Chinese Academy of Sciences, Guangzhou, China*

[2]*CSIRO Oceans and Atmosphere Flagship, Floreat, Western Australia, Australia*

[3]*Ocean University of China, Qingdao, China*

[4]*CSIRO Oceans and Atmosphere Flagship, Hobart, Tasmania, Australia*

Submitted to Scientific Reports
(2nd-round revised version)
Sep 2, 2015

Corresponding author address: Yan Du, State Key Laboratory of Tropical Oceanography, South China Sea Institute of Oceanology, 164 West Xingang Road, Guangzhou 510301, China. E-mail: duyan@scsio.ac.cn

Supplementary-Fig.1

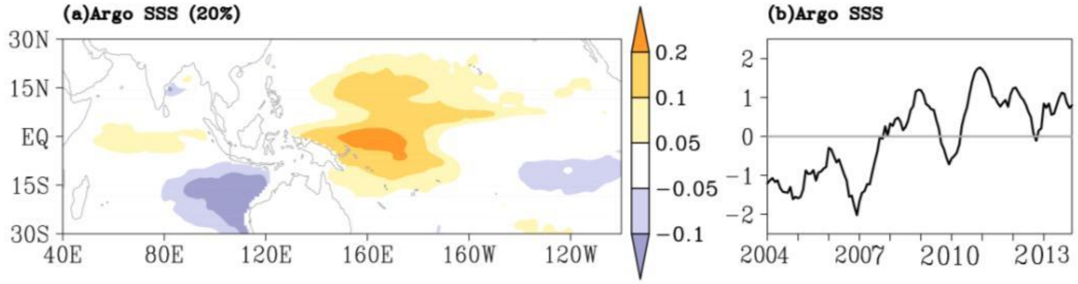

Supplementary-Fig.1. EOF analysis of Argo SSS. (**a**) First EOF mode (shaded, psu). (**b**) The principal component of Argo SSS anomaly. The figure is generated using GrADS.

Supplementary-Fig.2

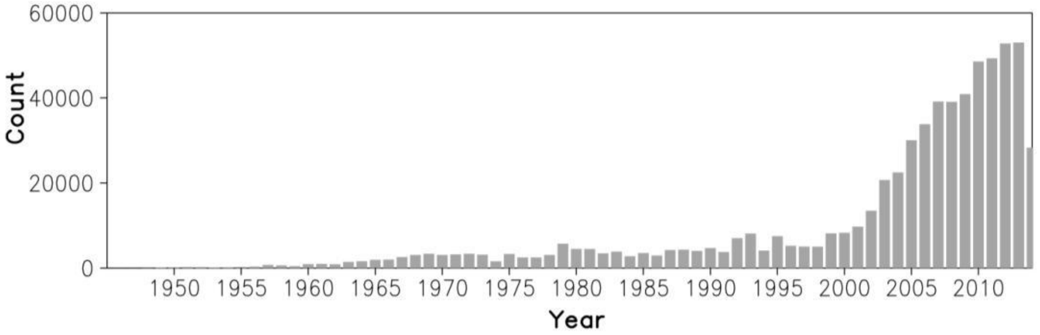

Supplementary-Fig.2. Annual counts of T/S profiles in the tropical Indo-Pacific in historical data (40 °-260 °E, 30 °S-30 °N). The figure is generated using GrADS.

Supplementary-Fig.3

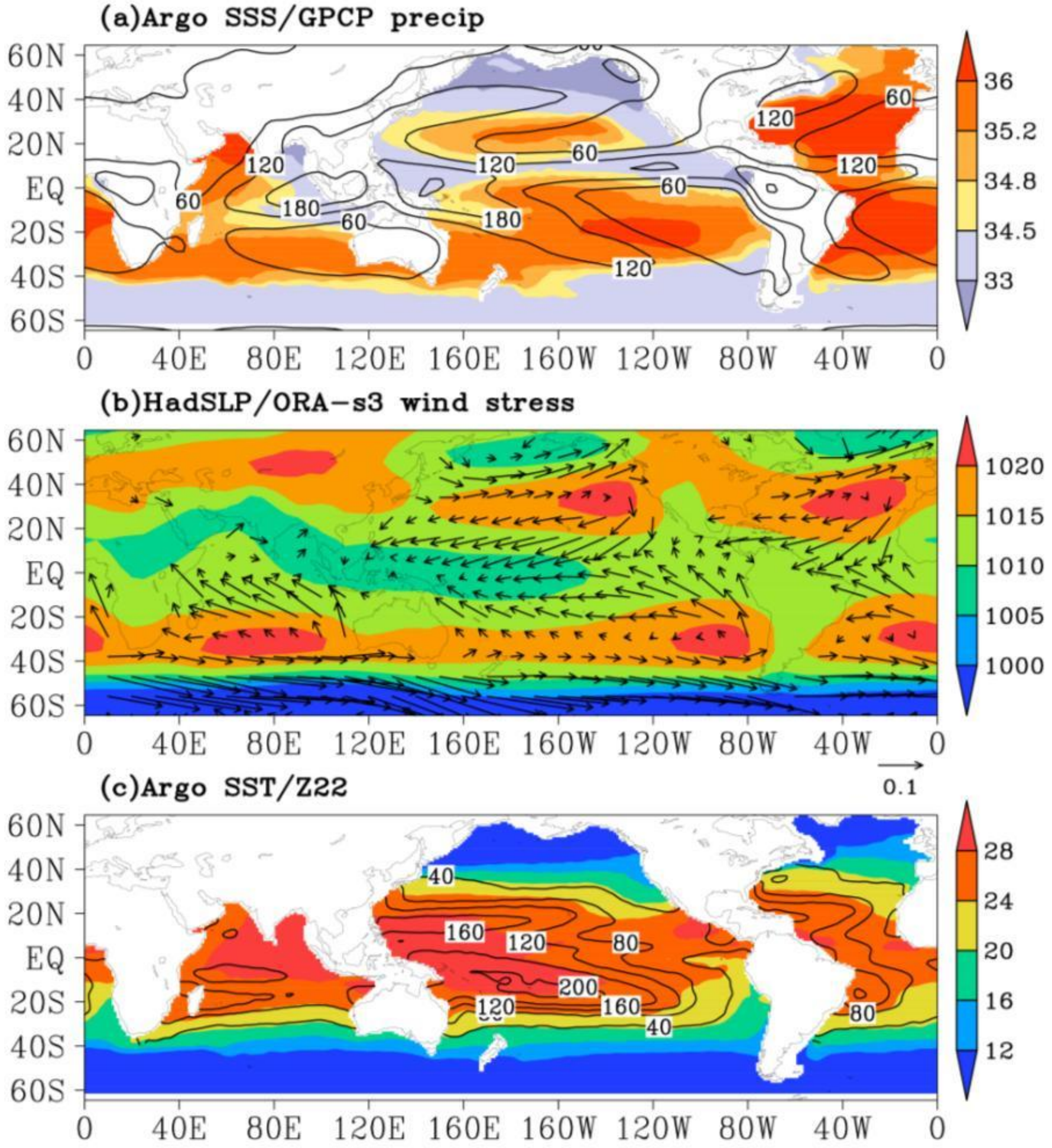

Supplementary-Fig.3. Annual mean of atmospheric and oceanic fields. (**a**) SSS (shaded, psu) and precipitation (contours, mm month^{-1}). (**b**) Sea level pressure (shaded, pa) and wind stress (vectors, kg m^{-1} s^{-2}). (**c**) SST (shaded, ℃) and depth of 22 ℃ (contours, m) in the global ocean between 65 °S to 65 °N. The figure is generated using GrADS.

Supplementary-Fig.4

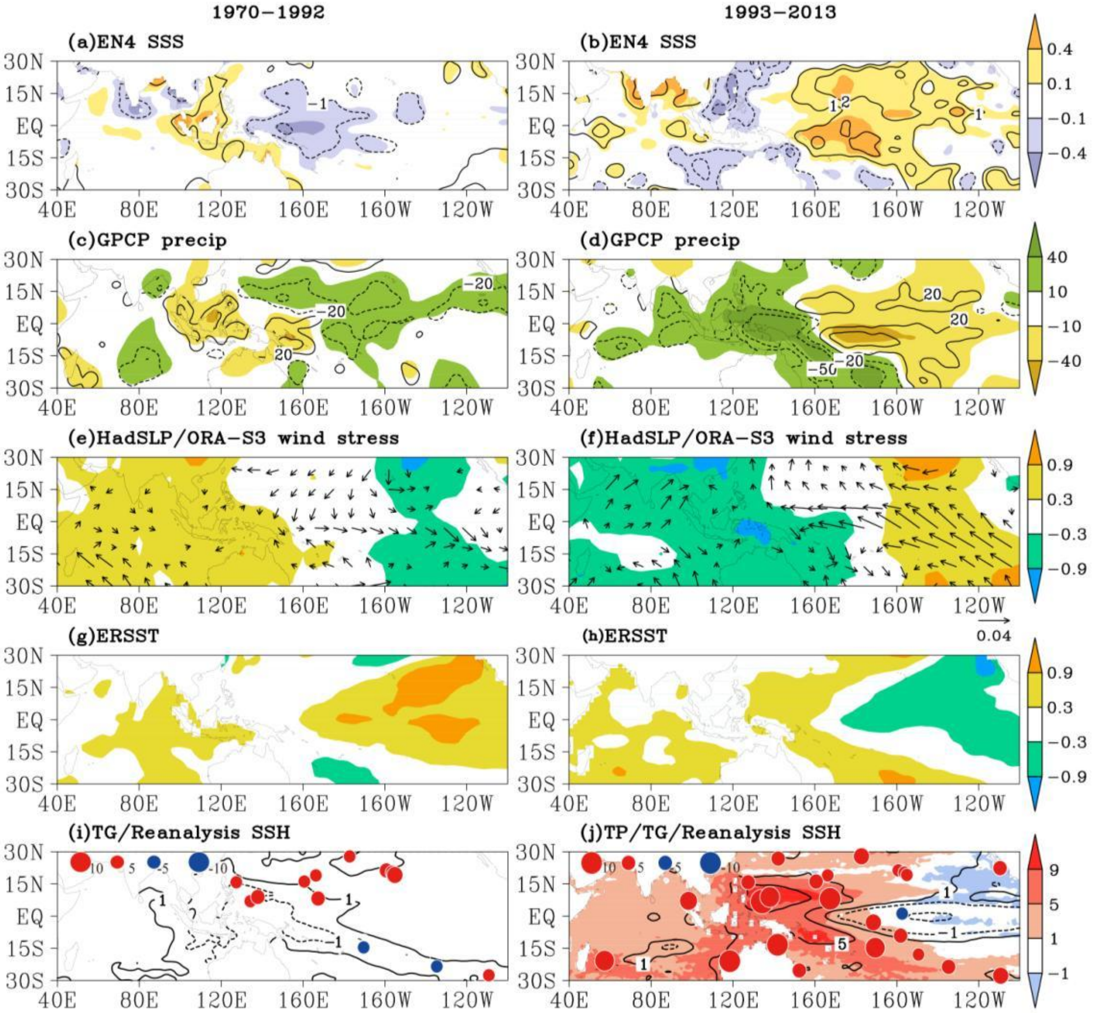

Supplementary-Fig.4. Linear trends of SSS, atmospheric fields, SST, and SSH before and after 1993. (**a**) SSS (shaded, psu), superimposed with ratio between the trend and the standard deviation of the detrended SSS (contour). (**c**) Precipitation (shaded, mm month^{-1}) and E-P (contour, mm month^{-1}). (**e**) Sea level pressure (shaded, pa) and wind stress (kg m^{-1} s^{-2}). (**g**) SST (℃). (**i**) Reconstructed sea level (contours, cm) and tide gauges sea level (solid circles, cm) for the period of 1970-1992. (**b**, **d**, **f**, **h**, and **j**) are

same as (**a**, **c**, **e**, **g**, and **i**), but for the period of 1993-2013. AVISO SSH trend (shaded, cm) is included in (**j**). The figure is generated using GrADS.
